# Sex differences in sucrose reinforcement in Long-Evans rats

**DOI:** 10.1186/s13293-022-00412-8

**Published:** 2022-01-11

**Authors:** Jeffrey W. Grimm, Katherine North, Madeleine Hopkins, Kyle Jiganti, Alex McCoy, Josef Šulc, Derek MacDougall, Frances Sauter

**Affiliations:** Department of Psychology and Program in Behavioral Neuroscience, Western Washington University, 516 High Street, Bellingham, WA 98225-9172 USA

**Keywords:** Craving, Cue-reactivity, Dopamine, Food, Motivation, Relapse, Sex-difference, Sucrose

## Abstract

**Background:**

There are sex differences in addiction behaviors. To develop a pre-clinical animal model to investigate this, the present study examined sex differences in sucrose taking and seeking using Long-Evans rats.

**Methods:**

Five experiments were conducted using separate groups of subjects. The first two examined sucrose or saccharin preference in two-bottle home cage choice tests. Experiment three assessed sucrose intake in a binge model with sucrose available in home cage bottles. Experiments four and five utilized operant-based procedures. In experiment four rats responded for sucrose on fixed and progressive ratio (FR, PR) schedules of reinforcement over a range of concentrations of sucrose. A final component of experiment four was measuring seeking in the absence of sucrose challenged with the dopamine D1 receptor antagonist SCH23390. Experiment five assessed responding for water on FR and PR schedules of reinforcement.

**Results:**

When accounting for body weight, female rats consumed more sucrose than water; but there was no sex difference in saccharin preference over a range of saccharin concentrations. When accounting for body weight, females consumed more sucrose than males in the binge model, and only females increased binge intake over 14 days of the study. Females responded at higher rates for sucrose under both FR and PR schedules of reinforcement. Females responded at higher rates in extinction (seeking); SCH23390 reduced sucrose seeking of both females and males. Females responded at higher rates for water on FR and PR schedules than males, although rates of responding were low and decreased over sessions.

**Conclusions:**

Across bottle-choice, binge intake, and operant procedures, female Long-Evans rats consumed more sucrose and responded at higher rates for sucrose. Although females also responded more for water, the vigor of responding did not explain the consistent sex difference in sucrose taking and seeking. The sex difference in sucrose taking was also not explained by sweet preference, as there was no sex difference in saccharin preference. These data provide a pre-clinical model to further evaluate sex differences in addiction behaviors and manipulations designed to reduce them.

**Supplementary Information:**

The online version contains supplementary material available at 10.1186/s13293-022-00412-8.

## Highlights

- Sucrose taking and seeking by rats provides a preclinical model of addiction behaviors.
- The present study describes sex differences in sucrose taking and seeking rat with female rats in several models, overall demonstrating more motivation by females to consume sucrose.
- Further examination of sex differences using these pre-clinical models could lead to better, sex-dependent treatment strategies for treating substance use disorder and disordered eating.

## Background

There are sex differences in some drug addiction behaviors including craving and relapse [1–4]. Furthermore, women are more vulnerable to addiction behaviors related to food. For example, women are more likely to have difficulty regulating food cravings compared to men [5] and eating disorders, characterized by high relapse potential, occur more often in women [6]. Women are also more likely to be diagnosed with severe and morbid obesity [6]. Although a number of economic and societal factors influence sex differences in drug-focused and eating behaviors, findings from animal models can provide critical insight into these differences as well.

Sugars are high-value reinforcers for humans and other species, including rats. Like humans, rats prefer sucrose-sweetened foods and will consume sucrose beyond caloric need [7, 8]. These and other behaviors including food binging [9] indicate that examination of the reinforcing effects of sucrose and other sugars provides insight into the profound effects of sugar on behavior and neurobiology, especially in the context of food and drugs of abuse [7, 8].

Sex differences in sweet preference by rats have been reported previously. Initial findings using a choice procedure identified females preferred saccharin more than males did [10]. Subsequent studies with sucrose have generally supported a sex difference with females preferring sucrose solutions more than males; however, caveats exist depending on methodology and stage of estrous [11, 12].

An extension of the choice procedure is the binge intake model, where rats are provided access to a sweet solution for either 12 or 24 h per day. Rats in the 12 h condition drink more sweet solution during the first hour of daily access [13]. Sex differences in binging of fat or a palatable mixture (fat + sugar) have been described, with females binging more than males [14, 15]. With sucrose alone as the reinforcer, females binged slightly more than males [13] and females binged more than males but this effect was observed only in Wistar-Kyoto, but not Wistar rats [16].

Finally, operant reinforcement models are quintessential for developing a deeper understanding of reinforcer-directed behavior. For example, depending on the reinforcement history and schedule of reinforcement, rate of responding on a lever for sucrose could be indicative of motivation to acquire the reinforcer, not just interest or preference. The fixed ratio (FR) schedule of reinforcement is often used to assess interest or preference, while the progressive ratio (PR) schedule of reinforcement is argued to provide a measure of motivation [17]. Responding in the absence of reinforcement, such as in extinction or for a cue paired previously with reinforcement, can be used to assess conditioned reinforcing properties of stimuli [18]. The latter approach is an established preclinical model of craving [19]. Currently, three studies have reported no significant sex differences in response-dependent sucrose self-administration using the FR schedule of reinforcement; all used sucrose pellets as the reinforcer [20–22]. PR results with sucrose alone have not yet been reported; however, in a study, where sucrose plus a preferred flavor was used as the reinforcer, females responded to higher break points than males [23]. Regarding sucrose cue reactivity, sex differences have been identified but findings both within and between laboratories are inconsistent. Specifically, females develop more Pavlovian approach behavior in response to a sucrose-associated cue compared to males [24] and respond more for a sucrose-paired cue compared to males [21]. However, the sex difference in responding for a sucrose-paired cue was not observed in a subsequent study from that same laboratory [22] and no sex difference was observed in the discriminative stimulus effects of a palatable pellet (fat, carb, protein combo) [25].

In summary, rat models have revealed sex differences in sucrose reinforcement with some exceptions. These exceptions might be traced to differences in data analyses (e.g., not correcting for body weight), or potential strain or age differences. The type of reinforcer (e.g., liquid vs. pellet) may also be consequential. To provide a more robust model for future research, we sought to examine sex differences in sucrose consumption, including motivation to consume, using the three general approaches outlined above (preference, binge, operant) in adult rats. Long-Evans rats served as subjects, a strain commonly used in basic research examining the neurobiology of drug and food reinforcement. This comprehensive approach was taken to allow us to better evaluate any sex differences we observed, as qualitative comparisons across the results using the various approaches would be made within the same laboratory with the same vivarium (source of subjects). We also included an initial assessment of potential sex differences in sucrose seeking challenged with the dopamine D1 receptor antagonist SCH23390. SCH23390 reduces reactivity to cues paired with a number of reinforcers including cocaine [26], propofol [27], nicotine [28], sucrose [29], and saccharin [30]. Thus, the SCH23390 challenge was a constructive replication of previous findings with sucrose plus an initial probe into potential sex differences in sucrose seeking mediated by dopamine neurotransmission.

## General methods

### Ethical approval

The experiments were conducted following welfare mandates and guidelines established by the National Institutes of Health [31], and were approved by the Western Washington University Institutional Animal Care and Use Committee.

### Subjects

Adult male and female Long-Evans rats (Simonsen-derived) were bred and raised in the WWU Psychology department vivarium. Breeding stock were produced from random mating of unrelated subjects with new outside breeders (sourced from Simonsen) brought in every 10 months. Rats were between 90 and 114 days at the start of each experiment. Rats were housed individually and placed in a reverse light cycle room (lights off 0700–1900 h) starting on post-natal day 70. Self-administration studies (Experiments 4 and 5) were conducted between 0900 and 1300 h 7 days a week. Food (Mazuri Rodent Pellets, Purina Mills Inc., Saint Louis, MO) was provided ad libitum in home cages and operant conditioning chambers (Experiments 4 and 5). Water was also provided ad libitum, except as noted below for the start of Experiment 4. Body weights were taken at the beginning of each Experiment and again either at the time of intake measurements (Experiments 1 and 2) or MWF for the duration of the study (Experiments 3–5). Separate animals were used in each Experiment.

### Apparatus

Cages used in all studies (home cages for Experiments 4 and 5) were clear plastic hanging cages (width × depth × height 20 × 30 × 20 cm; Lab Products Inc. (Seaford, DE, USA)). Water for home cages and operant conditioning chambers was supplied from a common source of filtered drinking water. Operant conditioning chambers (Experiments 4 and 5) were from Med Associates (30 × 20 × 24 cm; St. Albans, VT, USA). Each chamber included a retractable active lever, a stationary inactive lever for recording non-directed responding, four infrared photobeams to detect locomotion, a 2 kHz tone generator (15 dB over ambient noise), a white stimulus light above the retractable lever, and a red house light on the opposite wall. An infusion pump delivered sucrose into a receptacle to the right of the active lever. Chambers were enclosed in light and sound-attenuating cabinets with fans providing ventilation and white noise. Food and water were provided ad libitum, except water bottles were removed for the first day of operant conditioning.

### Statistical analyses

Analyses were conducted using Microsoft Excel (*t* tests) or IBM SPSS Statistics 28. Additional file 1 includes descriptive statistics and ANOVAs for reinforcer deliveries, inactive lever responses, and photobeam breaks in Experiments 4 and 5. Body weight adjusted reinforcers statistics for Experiment 4 are also provided in Additional file 1. For Experiments 1 and 2, preference was calculated over 1 g rat mass. In Experiment 3 intake was calculated over 1 g rat mass. In some instances, in the literature the denominator for preference is 100 g but we chose to present data for Experiments 1–3 over 1 g rat mass to be internally consistent with data presentation. Where warranted, post-hoc analyses were made using *t* tests with Šidák-corrected *p* values calculated incorporating the total number of comparisons made. As an indication of effect size, partial eta squared (*η*^2^) is provided for significant *F* tests and interactions and Cohen’s *d* is provided for significant *t* tests. The threshold for statistical significance was *p* < 0.05. In the text and figures, group averages are presented as mean ± standard error of the mean (SEM). *N* sizes and male and female body weights at the start of each experiment are provided for each Experiment.

## Specific methods and results: Experiments 1–5

### Experiment 1: sucrose preference

10 males and 10 females served as subjects. Weights at the start of the experiment were: males 392.7 ± 6.1 g, females 234.2 ± 4.4 g. Rat cages were supplied with two water bottles filled to 400 mL and sip tube access to a cage rack water supply was removed. Water used in all experiments originated from the same water filtration source. After 3 days of bottle access to water, the left side bottle was switched out with a bottle containing 10% sucrose. After 3 days, intake was assessed as mL fluid consumed. Rats were provided water in both bottles for 3 more days, followed by 2 days with the right-side bottle switched out with 10% sucrose. This shorter (48 h) intake assessment was implemented, because the first sucrose preference test period (72 h) resulted in 6 males consuming most of their sucrose (6 mL or less remaining in bottle). The shorter follow-up assessment resulted in 1 male consuming most of his sucrose.

The final 48 h preference data are reported adjusted by body weight (sucrose mL − water mL)/weight 1 g. Water was the intake in mL from the water bottle on that test day. Calculation of preference using this weight-adjusted formula has been used over the past several decades to characterize saccharin preference, especially to identify low vs. high saccharin-preferring phenotypes in rats [32]. We made one modification of the typical calculation: we used water intake during the choice period instead of water intake during the initial habituation to bottle access. This was implemented to be consistent with Experiment 2. In that experiment, the time between habituation to bottle access and the final choice measurements was over 2 weeks. We deemed it more reasonable to use water intake measures taken at the same time as saccharin measures. Preference scores were compared between males and females using a *t* test. Water intakes measured in mL/weight 1 g were compared between males and females using a *t* test.

**Fig. 1:**
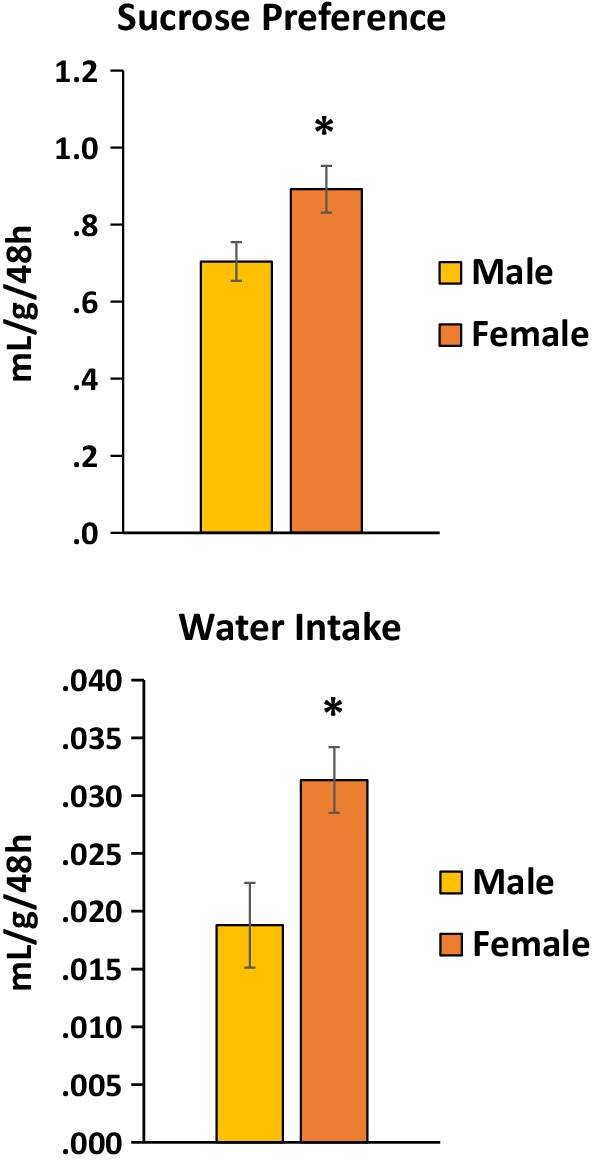
Sucrose preference females vs. males by body weight. Top: females preferred sucrose vs. water more than males, by body weight. *Females vs. males, *p* < 0.05. Bottom: females consumed more water than males, by body weight. *Females vs. males, *p* < 0.05

Over a 48-h period, females preferred sucrose more than males as measured by consumption of sucrose solution vs. water accounting for body weight *t*(18) = − 2.4, *p* < 0.05 (*d* = 1.2) (Fig. 1 top). Females also consumed more water during this period, by body weight *t*(18) = − 2.7, *p* < 0.01 (*d* = 1.2) (Fig. 1 bottom).

### Experiment 2: saccharin preference

10 males and 10 females served as subjects. Weights at the start of the experiment were: males 411.3 ± 9.6 g, females 244.1 ± 3.7 g. As with sucrose preference, rat cages were supplied with two water bottles and sip tube access to a cage rack water supply was removed. The general procedure followed that of [33]. After 3 days of bottle access to water, one bottle was switched out with a bottle containing saccharin (0, 0.075, 0.15, 0.3, or 0.6%) and intake was measured after 3 days of access. A switch of saccharin concentration occurred on four more occasions with rats receiving all concentrations counterbalanced and with the saccharin-containing solution alternated by side of the cage for each switch in concentration.

Preference data are reported adjusted by body weight as (saccharin mL − water mL)/weight 1 g. Water milliliter was the intake from the water bottle on that test day. Preference scores across the 5 concentrations were compared between males and females using two-way repeated measures analysis of variance (two-way RMANOVA). Water intake in mL/weight 1 g was compared between males and females across the five measures using two-way RMANOVA.

**Fig. 2:**
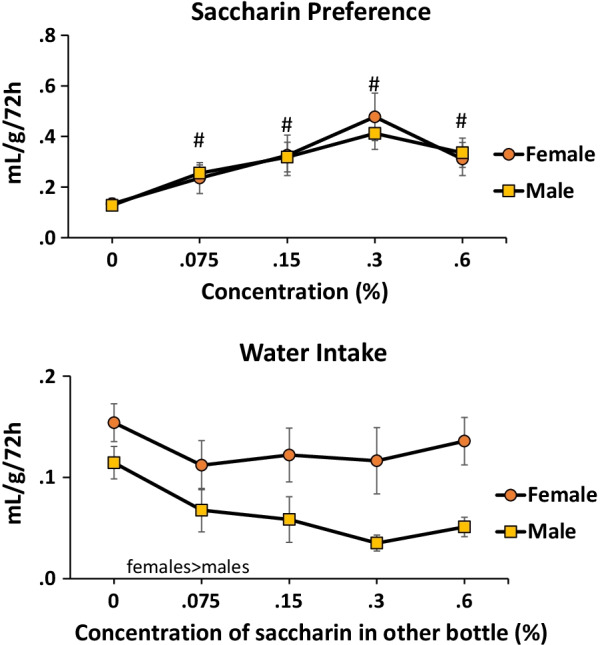
Saccharin preference females vs. males by body weight. Top: there was no sex difference in preference for saccharin across a range of concentrations. Regardless of sex, rats preferred all concentrations of saccharin over the 0% concentration. #vs. 0% concentration, *p* < 0.05 (females and males combined). Bottom: females consumed more water than males, by body weight, *p* < 0.05

There was no sex difference in saccharin preference across a range of saccharin concentrations (0, 0.075, 0.15, 0.3, 0.6%) with preference measured as consumption of saccharin solution vs. water accounting for body weight (Fig. 2 top). Both males and females preferred saccharin vs. water across the range of concentrations examined *F*(4,72) = 9.2, *p* < 0.001 (*η*^2^ = 0.3). Figure 2 top indicates saccharin preference by females vs. males by body weight. There was a sex difference in water intake across preference tests with females consuming more water *F*(1,18) = 12.5, *p* < 0.01 (*η*^2^ = 0.1). There was also a main effect of saccharin concentration in the other choice bottle *F*(4,72) = 2.5, *p* < 0.05 (*η*^2^ = 0.4), but post-hoc tests did not reveal any significant differences between concentrations (Fig. 2 bottom).

### Experiment 3: sucrose binge

24 males and 24 females served as subjects. Weights at the start of the experiment were: males 384.0 ± 6.2 g, females 230.5 ± 2.8 g. The approach for this experiment was to allow rats to have access to chow and 10% sucrose either 12 or 24 h per day. Similar to [13], a primary dependent measure was to examine how this manipulation affected sucrose intake in a 1-h daily access period (0800 h). For this Experiment, sip tube access to a cage rack water supply was retained to simplify the daily measures of sucrose solution and chow intake. Having only one bottle allowed chow to be placed in a hopper rather than on the floor of the cages. For days 1–3, a water bottle was supplied to habituate rats to a bottle. For days 4–18 all rats had access to a new 400 mL bottle of 10% sucrose at 0800 h. This was removed at 0900 h and replaced with a new bottle of 10% sucrose. At 2000 h the bottle was removed from the “12 h” rats. At the next 0800 h the bottle was removed from the “24 h” rats. Chow was removed for weighing and replaced with 100 g chow for the 24 h rats at 0800 h. For 12 h rats chow was removed and replaced at 2000 h. This procedure allowed for the following measures: 1 h sucrose intake for all rats (0800–0900 h), 12 h sucrose and chow intake for 12 h rats (0800–2000 h), and 24 h sucrose and chow intake for 24 h rats (0800–0800 h). Body weights were interpolated between MWF measures to allow conversion of daily intakes to a proportion of body weight.

Sucrose intake is reported adjusted by body weight as sucrose mL/weight 1 g. Intake across days of the study was compared between males and females and between sucrose access conditions using three-way RMANOVA. Separate analyses were conducted for overall daily intake (12 or 24 h) and binge test intake (1 h intake). Chow intake is reported adjusted by body weight as g/weight 1 g. Chow intake across days of the study was compared between males and females and between sucrose access conditions using three-way RMANOVA.

**Fig. 3:**
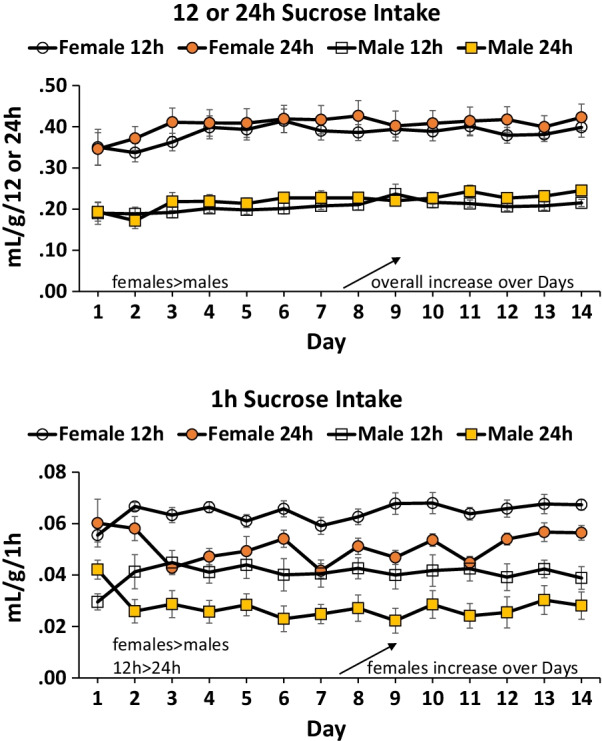
Binge data 12 vs. 24 h and 1 h intake by access condition females vs. males. Females consumed more sucrose than males across days of the study. Top: overall intake (12 or 24 h) increased over days for both males and females. Bottom: 1 h intake was greater by rats with a history of 12 vs. 24 h sucrose access, indicative of binge intake. 1 h intake increased over days for females only

Females consumed more sucrose than males over the 14 days of the Experiment during 12 and 24 h access (Fig. 3 top) and during the 1 h access (Fig. 3 bottom) periods. There was a significant effect of sex for 12 and 24 h access *F*(1,44) = 84.8, *p* < 0.001 (*η*^2^ = 0.7), and for 1 h access *F*(1,44) = 69.2, *p* < 0.001 (*η*^2^ = 0.6). Intake during 12 or 24 h access increased across the 14 days of the experiment for both males and females *F*(13,572) = 6.2, *p* < 0.001 (*η*^2^ = 0.1). Intake during 1 h access increased across the 14 days for females, time × sex *F*(13,572) = 1.9, *p* < 0.05 (*η*^2^ = 0.04). Intake during 1 h access was greater by rats with 12 vs. 24 access. This was indicated by a significant main effect of access condition *F*(1,44) = 22.2, *p* < 0.001 (*η*^2^ = 0.3) and time × access condition *F*(13,572) = 3.7, *p* < 0.001 (*η*^2^ = 0.08). The interaction is explained by no difference between conditions during the initial test sessions; the difference develops over time (Fig. 3 bottom).

Chow intake (data not shown) decreased by 20.7% across the 14 days of the experiment *F*(13,572) = 14.4, *p* < 0.001 (*η*^2^ = 0.2) but also varied according to sex or sucrose intake condition. There was a time × sex interaction *F*(13,572) = 2.9, *p* < 0.001 (*η*^2^ = 0.06), and a time × condition interaction *F*(13,572) = 2.9, *p* < 0.001 (*η*^2^ = 0.06). For time × sex, females consumed an average of 13.9% more chow over the first 3 days of the experiment with similar consumption between males and females thereafter. For time × condition, consumption was 17.4% greater on day 1 of the experiment for 24 h sucrose access rats, but for the rest of the experiment consumption was similar between access conditions.

### Experiment 4: response-dependent sucrose

24 males and 24 females served as subjects. Weights at the start of the experiment were: males 363.4 ± 4.5 g, females 219.7 ± 3.6 g. To encourage acquisition of lever pressing for sucrose solution, water was removed from home cages for the 17 h prior to the first day of training. Operant conditioning sessions began with extension of the active lever and illumination of the red house light. Rats first self-administered in 10 daily 2 h sessions 0.2 mL of 10% sucrose on a FR1 schedule of reinforcement, with sucrose delivery contingent on an active lever response. Sucrose delivery was paired with a 5 s tone + white light cue. There was a 40 s timeout before availability of the next reinforcer. Rats were then randomly assorted to two different groups to be tested with either 0, 3.75, 7.5 (low range) or 7.5, 15, 30% (high range) concentrations of sucrose. Concentrations were tested in counterbalanced order with 3 days of 10% sucrose available in between each test. Following a subsequent FR training day, rats trained with 10% sucrose on a progressive ratio (PR) schedule for 7 days. Sessions began with extension of the active lever and illumination of the red house light. The reinforcement contingency for responding on the active lever then escalated according to [34] with the progression 1, 2, 4, 6, 9, 12, 16, 20, 28, 36, 48, 63, 83, 110, 145, 191, 251, 331, 437, 575, 759, 999, 999 (repeat). Following a reinforced response, the active lever retracted for the duration of the tone + white light cue (5 s) and 0.4 mL sucrose delivery (total of 6.1 s). Sessions ended after 3 h or 30 min of no active lever responses. After training, rats were tested on PR with the aforementioned concentrations, with 3 days of 10% sucrose (PR) in between each test. Finally, rats trained again on FR for 3 days, then had 3 extinction tests with 3 FR re-training sessions in between each test. Extinction tests were identical to training sessions except sucrose was not available. Prior to each extinction test, rats were pretreated with the dopamine D1 antagonist SCH23390 (Sigma, St. Louis, MO, USA) (0, 1, 10 μg/kg IP, 15-min pretreatment, counterbalanced). Saline was the vehicle. As a handling control, rats received IP injections of saline the two afternoons preceding the first drug challenge.

Operant conditioning data are reported as the number of active lever presses in each session. Training data for the two concentration–response cohorts (low range, high range) were combined after initial three-way RMANOVA of the six dependent measures (days 1 and 10 FR, FR day between FR and PR training, days 1 and 7 PR, and day 3 of FR training prior to FR extinction testing) revealed no cohort effect. Training FR (10 days) and PR (7 days) responses were compared between males and females using two-way RMANOVA. Testing FR and PR responses were compared between males and females using two-way RMANOVA with the two concentration–response cohorts analyzed separately. FR extinction testing data (SCH23390 challenge) were compared between males and females using two-way RMANOVA with the previously tested concentration–response cohorts combined. FR extinction testing data were analyzed a second time using day 10 of FR training active lever responding as a covariate (ANCOVA).

Active lever responses are reported here. Other dependent measures (sucrose deliveries, sucrose deliveries considering body weight, inactive lever responses, photobeam breaks) are presented in Additional file 1. As noted above, the training and extinction data of the two concentration–response cohorts were combined after initial three-way RMANOVA of the six dependent measures (days 1 and 10 FR, FR day between FR and PR training, days 1 and 7 PR, and day 3 of FR training prior to FR extinction testing) revealed no cohort effect.

#### FR training

**Fig. 4:**
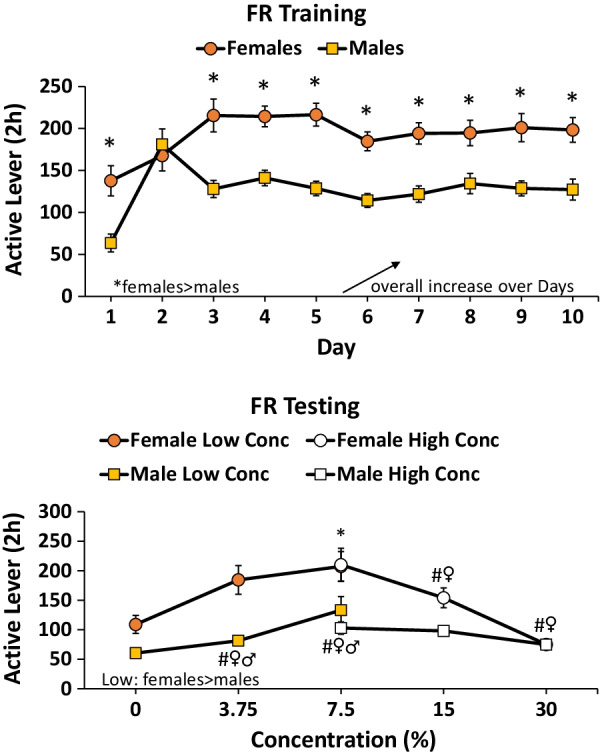
Sucrose FR training and testing females vs. males. Top: females responded more for sucrose than males and responding for both females and males increased over days of training. Bottom: across the low range of concentrations, females responded more than males and male and females responded more for 3.75 and 7.5% concentrations of sucrose compared to 0% (#vs. 0% concentration, *p* < 0.05). Across the high range of concentrations, males and females differed at 7.5% (*p < 0.05) and only females decreased responding at the 15 and 30% concentrations vs. 7.5% (#vs. 7.5%, *p* < 0.05)

Females responded at a higher rate than males *F*(1,46) = 33.6, *p* < 0.001 (*η*^2^ = 0.4) and this effect was consistent after the second day of training (time × sex interaction) *F*(9,414) = 3.3, *p* < 0.01 (*η*^2^ = 0.07). Responding of both males and females increased over the 10 days of training *F*(9,414) = 7.9, *p* < 0.001 (*η*^2^ = 0.1) (Fig. 4 top).

#### FR testing

For the low range (0, 3.75, 7.5%), there were 14 male and 12 female subjects. Females responded more than males overall *F*(1,24) = 13.8, *p* < 0.01 (*η*^2^ = 0.4). Both sexes responded more for the 3.75 and 7.5% sucrose vs. water (0%) *F*(2,48) = 21.0, *p* < 0.001 (*η*^2^ = 0.5) (Fig. 4 bottom). For the high range (7.5, 15, 30%), there were 10 male and 12 female subjects. Females responded more than males *F*(1,20) = 15.9, *p* < 0.01 (*η*^2^ = 0.4), only at the 7.5 and 15% concentrations, interaction *F*(2,40) = 6.2, *p* < 0.01 (*η*^2^ = 0.2) (see post-hoc tests on Fig. 4 bottom). Males were not as sensitive to changing concentrations of sucrose. For the high range, males did not differ in responding across the three concentrations (RMANOVA n.s.). In contrast, following a significant RMANOVA for females *F*(2,22) = 13.8, *p* < 0.001 (*η*^2^ = 0.6) there were significant post hocs comparing 7.5 vs. 30% and 15 vs. 30% (Fig. 4 bottom).

#### PR training

**Fig. 5:**
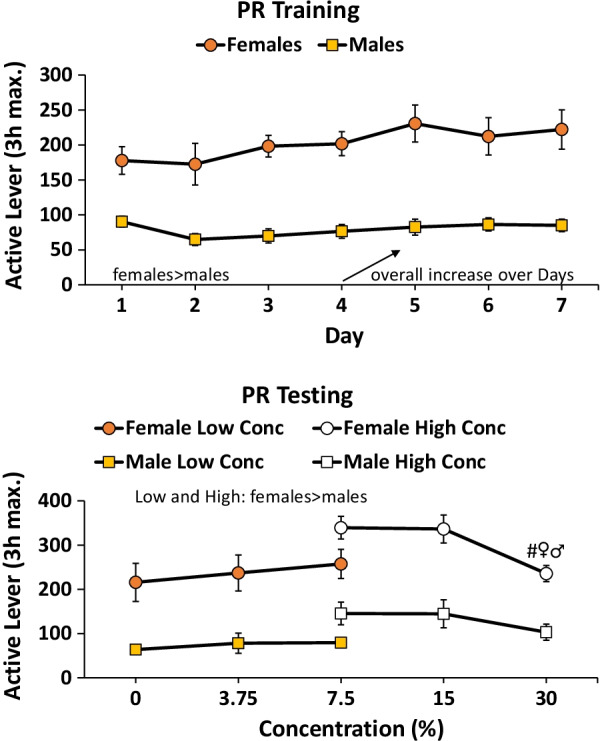
Sucrose PR training and testing females vs. males. Females responded for sucrose more than males during training and testing. For training (top), responding by males and females increased over days of training. For testing (bottom), the only concentration-dependent effect was a decrease in responding at the 30 vs. 7.5% concentration only, for both females and males (#vs. 7.5%, *p* < 0.05)

Females responded at a higher rate than males *F*(1,46) = 32.2, *p* < 0.001 (*η*^2^ = 0.4). Responding of both males and females increased over the 7 days of training *F*(6,276) = 3.1, *p* < 0.01 (*η*^2^ = 0.06) (Fig. 5 top).

#### PR testing

For the low range (0, 3.75, 7.5%), there were 14 male and 12 female subjects. Females responded more than males overall *F*(1,24) = 29.1, *p* < 0.001 (*η*^2^ = 0.5). There were no concentration-dependent effects with sucrose. For the high range (7.5, 15, 30%), there were 10 male and 12 female subjects. Females responded more than males overall *F*(1,20) = 10.9, *p* < 0.01 (*η*^2^ = 0.4). There was a significant effect of sucrose concentration *F*(2,40) = 4.3, *p* < 0.05 (*η*^2^ = 0.2) with a significant post-hoc test comparing 7.5 vs. 30% (Fig. 5 bottom).

#### SCH23390 challenge when responding in extinction

**Fig. 6:**
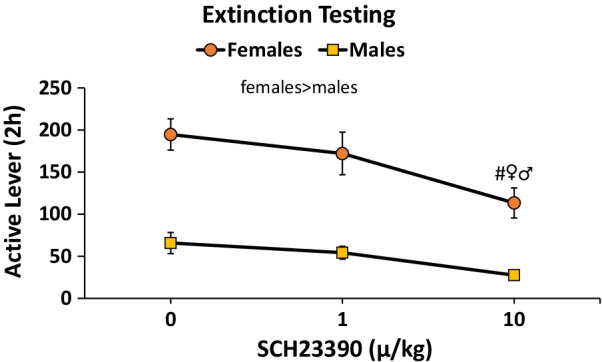
Sucrose extinction and D1 antagonist females vs. males. Females responded more than males. SCH23390 reduced responding at the high dose (#vs. 0 dose, *p* < 0.05 females and males combined)

**Fig. 7:**
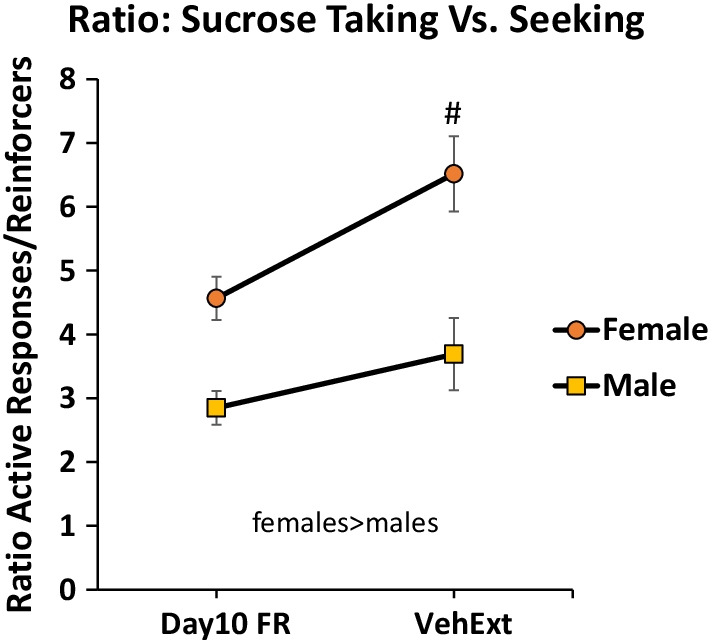
Sucrose taking vs. seeking females vs. males. Females responded at higher ratios of active lever responses to reinforcers (sucrose on FR day 10, tone + light cue on Veh Extinction) than males; females, but not males, had relatively higher ratios on the Veh Extinction day, #vs. FR day 10, *p* < 0.05

Females responded at a higher rate in extinction *F*(1,46) = 71.0, *p* < 0.001 (*η*^2^ = 0.6). SCH23390 pretreatment reduced responding *F*(2,92) = 7.1, *p* < 0.01 (*η*^2^ = 0.1) with a significant post-hoc test comparing 0 vs. the 10 μg/kg dose (Fig. 6). The fact that females responded at a higher rate in extinction is confounded by their higher rate of responding when responding for sucrose. ANCOVA analysis revealed that the extinction sex difference was not accounted for by the higher rate of responding during training; the significant sex difference in extinction responding remained *F*(1,45) = 45.5, *p* < 0.001 (*η*^2^ = 0.5). ANCOVA results support a sex difference in extinction responding despite higher reinforced responding during training. To address the question of a sex difference in extinction or, arguably, “cue-reinforced” responding further, we calculated ratios of active lever responding to sucrose deliveries on day 10 of training and ratio of active lever responding to cue deliveries during the vehicle pretreatment extinction test day for all rats. We then compared male and female rats with these ratios using two-way RMANOVA. Females had larger ratios overall *F*(1,46) = 25.5, *p* < 0.001 (*η*^2^ = 0.4). The ratios for male rats did not differ between training (responding for sucrose; 2.8 ± 0.3) and extinction testing (responding for sucrose-paired cue; 3.3 ± 0.4); however, ratios for females increased comparing training to extinction testing (from 4.6 ± 0.3 to 6.5 ± 0.6) with a significant post-hoc comparison following a significant interaction *F*(1,44) = 5.3, *p* < 0.05 (*η*^2^ = 0.1). In summary, males responded with a similar ratio of responses to reinforcers for sucrose itself or a sucrose-paired cue. Females overall responded at a higher rate for both sucrose and a sucrose-paired cue than males. Females also responded approximately 1.4 times as much for a sucrose-paired cue compared to sucrose itself, demonstrating that females are more cue-reactive than males (Fig. 7).

### Experiment 5: response-dependent water

13 males and 13 females served as subjects. Weights at the start of the experiment were: males 414.0 ± 14.2 g, females 222.6 ± 3.5 g. Rats responded for water (0.2 mL) on a FR (10 days) and then PR (0.4 mL) schedule (7 days). Training conditions were identical to response-dependent sucrose self-administration except the reinforcer was water. Following PR training, rats responded again on the FR for 3 days followed by 1 day of FR with no water reinforcement (extinction). This experiment was conducted as a comparison to Experiment 4, where rats responded for sucrose. The aim was to quantify how much rats respond for a reinforcer assumed to be of low value; such responding might be indicative of “baseline” responding for the novelty of reinforcer delivery.

Operant conditioning data are reported as the number of active lever presses in each session. FR training, PR training, and the final FR re-training plus extinction test responses were compared between males and females using two-way RMANOVA.

**Fig. 8:**
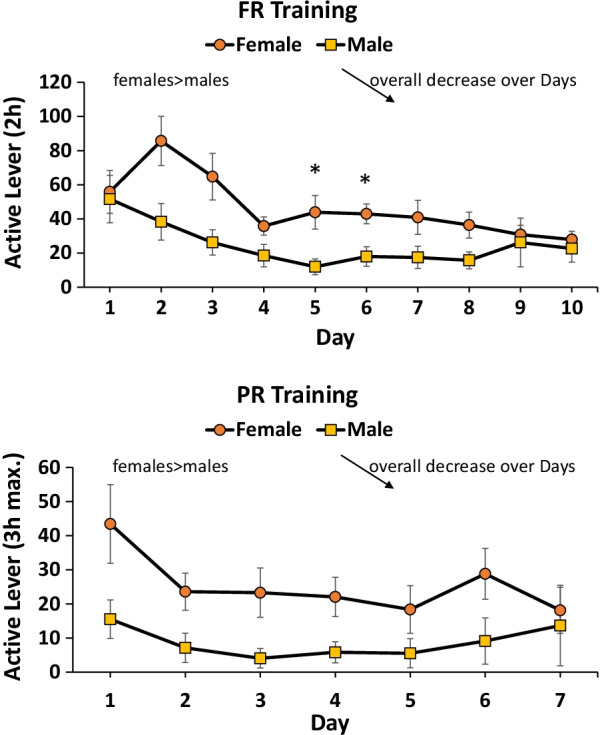
Water taking; females vs. males. For FR training (top), females responded more than males on days 5 and 6 (*p < 0.05). Overall, responding decreased over days for both females and males. For PR training (bottom), females responded more than males. Both females and males decreased responding over 7 days

Active lever responses are reported here. Other dependent measures (liquid reinforcer deliveries, inactive lever responses, photobeam breaks) are presented in Additional file 1. Female rats responded at a higher rate than males for water on the FR schedule of reinforcement *F*(1,24) = 5.5, *p* < 0.05 (*η*^2^ = 0.2). There was an effect of days of training *F*(9,216) = 7.3, *p* < 0.001 (*η*^2^ = 0.2) and a significant time × sex interaction *F*(9,216) = 2.3, *p* < 0.05 (*η*^2^ = 0.1), illustrated in Fig. 8 as females responding more than males over the first days of training, but the difference being negligible by day 10 of training (Fig. 8). PR training was similar in this profile (Fig. 8) with a main effect of sex *F*(1,24) = 4.4, *p* < 0.05 (*η*^2^ = 0.2) and time *F*(6,144) = 3.6, *p* < 0.01 (*η*^2^ = 0.1) but there was no significant interaction. In summary for PR, females responded at a higher rate than males and overall responding decreased over the 7 days of training for both males and females. Water deliveries, inactive lever responses, and locomotor activity followed trajectories similar to active lever responding (Additional file 1).

For the FR re-baseline and subsequent extinction test after completing the 7 days of PR training, there were no significant effects. At this point in the study, response rates for water were low and did not differ between males and females. In addition, when responding for the water-paired cue alone (extinction test), rate of responding did not differ between males and females (Additional file 1).

## General discussion

In summary, we observed sex differences in sucrose reinforcement in three assays. Females preferred sucrose over water more than males, females consumed more sucrose than males when available via bottles or after a lever response, and females responded at a higher rate for sucrose-paired cues in extinction conditions. There were no sex differences in saccharin preference, nor in extinction responding following a challenge with the dopamine D1 receptor antagonist SCH23390.

### Sweet preference

Female rats preferred 10% sucrose over water to a greater degree than male rats. This preference was observed using simple bottle choice considering body weight (Experiment 1) (Fig. 1) and with greater sucrose intake in the binge study (Experiment 3) (Fig. 3). Greater preference of sucrose by female vs. male rats has only inconsistently been reported previously, with sex differences more likely to be observed in binge access studies (see below). In a comprehensive examination of tastant preference across 14 rat strains, sex differences in sucrose preference were negligible including for Long-Evans rats [35]. These comparisons were made using sucrose intake as a percentage of total fluid (sucrose + water). Body weight was not considered in these comparisons, despite the substantial sexual dimorphism in body mass observed in many strains of adult rats. In contrast, many studies describing saccharin preference consider body weight. For example, [32] describes a calculation of saccharin preference score similar to that used in the present study, in their case to differentiate low vs. high saccharin preferring phenotypes. Incorporating body weight into the preference calculation emphasizes the importance of the absolute intake of the sweet substance for a specific individual; in effect the measure is a mixture of preference and avidity. This approach to quantifying preference in some studies, but not others, may also explain a lack of sex difference in sucrose preference in other studies (e.g., [36–38]). For the present study, incorporating body weight also allows within-study qualitative comparison across Experiments 1,2,3.

We did not find a sex difference in saccharin preference (Fig. 2) using a calculation similar to the “phenotype” calculation [32]. This was surprising as female rodents have consistently been reported to prefer saccharin to a higher degree than males starting with [10]. However, as noted above with a simple preference measure (percent sweet intake of total liquid intake) there were no robust sex differences in sucrose preference comparing 14 strains of rats; there were also no differences in saccharin preference [35]. It is, therefore, important to consider how preference is calculated, possibly the strain of rat examined, and other variables including age. For example, [39] reported greater preference for sucrose by females vs. males using a simple percentage score but only for older adult (tested at postnatal day 140) not younger adult (postnatal day 70) or adolescent (postnatal day 42) rats. These discrepancies prevent us from making a definitive, cross-strains conclusion regarding sex differences in sweet preference.

Interpreting a sex difference in preference, as we observed for sucrose, is complicated in that several factors could influence preference. Taste reactivity or perception could affect intake. Adult male and female rats were recently observed to not differ in sucrose taste-reactivity [40]. Research on taste perception has included examination of sex differences at the level of the taste buds and projections to the brain stem via the glossopharyngeal nerve [12]. This review of the literature concluded female rats may be more sensitive to some tastes, including sweet tastes, at the level of the taste buds and brain stem efferents and that these effects can vary according to estrous cycle related hormones (and see [41]). In addition, [11] described more intense licking for low concentrations of sucrose by female vs. male rats, and sex and estrous cycle effects on sweet taste reactivity in rats [42]. These last findings are somewhat translatable to humans as perception of sweet intensity over a range of concentrations of sucrose was stronger for female participants [43]. Whether these findings are important for actual sucrose preference and intake is unclear. For example, [44] reported in humans that response to sucrose taste did not predict sucrose preference. In addition, it has been argued that the relationship between taste preference and food intake is difficult to quantify in humans [45]. However, perhaps, more important for the translatability of the present results (female > males sucrose preference) is that women consume more sweet and fat calories [46], are more drawn to sweets than men [47] and, across two cultures, report more craving for sweets than men [48]. These behaviors (intake, craving) are arguably modeled better by Experiments 3 and 4 of the present study: sucrose binge intake, and sucrose self-administration. That being said, it is important to state that sucrose intake and craving are by themselves not necessarily pathological; for humans, only in certain situations do these behaviors become problematic. The pre-clinical models capitalize on the ability to examine these behaviors in great detail in controlled conditions.

### Sucrose binge

Females rats consumed more sucrose compared to males which is consistent with other studies of sucrose bottle access over many days [24, 49]. Both males and females increased sucrose intake over the 14 days of the experiment, perhaps suggestive of an escalation in sucrose consumption although this is confounded with an expected accelerating acquisition curve. Binge intake in this model is indicated when rats that have had 12 h access to sucrose consume significantly more sucrose in a 1 h consumption test than rats with 24 h access to sucrose. In the present study, binge intake was apparent by both male and female rats. While there was no main effect of sex for binge intake during the 1 h consumption measure, there was a sex difference in binge intake “escalation” in the 1 h access measure, where only females slightly increased consumption over days if they were in the 12 h but not 24 h access condition (Fig. 3).

There are few studies in the literature to compare the present results, as sex differences in binge intake with rats has largely been with rats consuming fat or fat + sugar combinations. Of those with sucrose only binge intake, [13] reported a moderate sex effect with 1 h intake in 12 h rats with females with greater intake than males on some days of the experiment. As noted above, we observed an increase in 1 h intake across days for females only; [13] observed an increase for both sexes. This lab [50] also found, in a study with only females, binging was more pronounced in proestrus indicating that even if a sex difference in binge intake is moderate, the behavior is related to estrous cycle. Finally, [16] examined sex differences in binge intake across two rat strains and found a sex difference with females showing more binge intake, but only in Wistar-Kyoto, not Wistar rats. In contrast to these somewhat inconsistent findings, female rats showed greater fat or fat + sugar binge behavior [14, 15]. Further research is required to better elucidate the effect size of sex differences in sucrose binging; this may require further examination of strain differences. Studies might also contrast sucrose binging with fat binging to explore why the sex difference in fat binging is more robust. For example, in operant studies fat + sucrose is more reinforcing than either fat or sucrose alone [51]. In any case, both male and female rats demonstrate sucrose binging with some suggestion of more binging by females. These results do not generally correlate with clinical studies reporting higher food binge behavior by women [52]. However, clinical prevalence rates may be distorted by reporting constrained by ethnic/racial disparities in individuals seeking support for eating disorders [52].

### Operant self-administration

As noted above, operant paradigms may provide more robust models of sucrose consumption behavior. This includes studies of responding for reinforcement (taking) and responding in the absence of reinforcement (seeking). The present study evaluated sucrose taking behavior using two schedules of reinforcement, the FR and PR, with the latter argued to be a reasonable measure of motivation to acquire a reinforcer. Using the PR, reinforcing efficacy can be determined [17]. Active lever responding, indirectly tied to reinforcement, is a common measure of operant response behavior. For the present study, all response data in figures are reported as active lever responses. Active lever responses are not conducive to body weight adjustment, although the number of earned reinforcers would be (see [23]). Analysis of body weight adjusted sucrose self-administration earned reinforcers (Additional file 1) resulted in the same general findings as for active lever responses, although the sex differences were exaggerated.

Our robust sex differences across FR (Fig. 4) and PR (Fig. 5) schedules of reinforcement, and across some concentrations of sucrose, contrast with previous findings reporting no sex differences [20–22]. These previous studies all used sucrose pellets as reinforcers, compared to sucrose solution used in the present study. A side-by-side comparison of responding for pellets vs. sucrose solution could reveal whether this is a pellet vs. liquid solution issue, or an issue of differences in laboratory procedures. As the sex differences observed thus far are with liquid (present study) but not pellets, it is likely some aspect of the dry vs. liquid reinforcement is most important. Further study on this issue might include examination of palatability and possibly pharmacokinetic implications of dry vs. liquid sucrose reinforcement.

As with binge research, sex differences in palatable food taking and seeking has thus far included studies with sucrose, sucrose + flavor, fat, or fat + carbohydrate combinations. Ref. [53] examined sex differences in an operant paradigm with within-session increases in response requirements designed to assess intake depending on demand. Demand at zero (null) cost was estimated. Female rats were estimated to have a higher demand for sucrose and sucrose + fat reinforcement at null cost, but not at higher costs. It is notable that active lever responding on a FR 1 was not different between males and females, but the null estimate that incorporates body weight revealed the significant sex difference at the estimated null cost. Finally, in a study that incorporated PR intake, females had greater intake vs. males of a sucrose + flavor reinforcer [23]. This study, referred to above, also required normalization by body weight (reported as kcals/g^2/3^) to reveal the sex difference.

Extinction responding (cue-reactivity) was more pronounced in females vs. males in the present study. In Fig. 6, females are shown to respond over 3 times as much as males in the vehicle dose condition of the SCH23390 study. Overall, these values (males and females) are larger than what we typically see in sucrose cue-reactivity studies. This is likely due to the fact that the rats had just been responding on the response demanding PR schedule of reinforcement as part of Experiment 4. The present results somewhat concur with previous studies of sex differences in conditioned responding to a sucrose-paired cue. Ref. [24] reported females develop more Pavlovian approach behavior compared to males in response to a sucrose-associated cue. Ref. [21] found that females respond more for a sucrose-paired cue compared to males in an operant conditioning procedure similar to ours. Interestingly, that same group did not observe a sex difference in a subsequent constructive replication [22]. In addition, there was no sex difference in discriminative stimulus effects (CS+) of a palatable pellet (fat, carb, protein combo) [25]. As with other inconsistencies reported above, the differences in procedures, strains, and possibly age of subjects could account for the lack of consistent sex differences in sucrose seeking across studies. What is most salient with the present results are the robust sex differences observed.

Finally, SCH23390 decreased responding of both males and females to a similar extent (Fig. 6). The ability of this dopamine D1 antagonist to reduce cue-reactivity has been demonstrated for several drug and non-drug reinforcers including sucrose [26–30]. Ours are the first results we are aware of where both male and female rats were challenged with SCH23390 under sucrose self-administration extinction conditions. These findings indicate that the sex difference in responding is not specifically tied to D1 receptors, but, furthermore, that D1 antagonism is equally effective in males and females at reducing sucrose seeking in a preclinical model of craving.

The higher response rate by females during sucrose FR and PR training, and the higher, but diminished difference over training, response rate for water by females (Fig. 8) introduces a potential confound for interpreting both the sex differences in sucrose training but also cue-reactivity. As noted above, using training response rate as a covariate did not affect the ANOVA outcome of a sex difference in extinction responding. Furthermore, our analysis of the ratio between active lever and reinforcer delivery (sucrose-paired cue delivery for extinction testing) to normalize responding between sexes indicated females responded more for the sucrose-paired cue than males (Fig. 7). We do not know what rats are responding for in our water self-administration procedure. The rats have ad libitum access to food and water. It could be the rats are responding for the novelty of water and/or cue presentations, as described previously with rats responding for a presentation of a light stimulus [54]. Nonetheless, the magnitude of responding for water does not appear to account for the sex differences in operant responding for sucrose. First, the sex difference in response rate for water (FR, then PR) disappeared over the 10 (FR) or 7 (PR) days of training. Second, we subtracted the grand means of all training days for sucrose for males from those for females and then the same was done for water. Grand means for training were: FR sucrose females–males = 65.6 active lever presses, FR water females–males = 21.8, PR sucrose females–males = 122.8, and PR water females–males = 16.7. A relative comparison was then made between males and females in terms of the magnitude of the difference between males and females responding for sucrose or water. The FR difference between males and females was 300% more for sugar vs. for water, while the PR difference between males and females was 736% more for sugar vs. for water. In summary, females responded for water at higher rates than males but the effect was transient and rates for both males and females were orders of magnitude below that of responding for sucrose.

Even so, in nearly every component of the present set of experiments, where water intake was measured, water intake varied by sex. Female rats in Experiment 1 (sucrose preference), Experiment 2 (saccharin preference), and Experiment 5 (water self-administration) drank or responded for more water than males. More intake by female rats has been reported in preference studies, for studies that report water intake (e.g., [55]). Further study on this effect is required as water homeostasis could affect variables key to interpreting sex differences in animal models including food intake and drug pharmacokinetics.

### Other potential confounds

Inactive lever and photobeam breaks (locomotor) data ANOVA results for Experiments 4 (sucrose self-administration) and 5 (water self-administration) are provided in Additional file 1. A general finding was that female rats were more “active”, reflected as increased inactive lever presses and photobeam breaks. It is not clear how this background of increased activity could account for the sex differences in sucrose taking and seeking observed in the present study. Female rats have been noted to move about more than male rats in previous studies. For example, female rats have a greater locomotor response to a novel environment [22]. It is important to note that these measures were higher for females in both the sucrose and water self-administration experiments in the present study. That is, elevated inactive lever and photobeam breaks may be indicative of background activity not necessarily connected to motivated, or even impulsive behavior. General increased activity and response rate is important to consider for future studies.

## Perspectives and significance

The present study provides a baseline for further investigation of sex differences in sucrose taking and seeking in rats. Extending the generality across species, these results mostly parallel a recent study with mice as subjects [56]. Further studies are required to evaluate a potential role of gonadal hormones in these sex differences, and to explore the neurobiology of sucrose taking and seeking in males and females. For example, mesolimbic estrogen/dopamine interactions [57] have been described extensively in the literature and mesocorticolimbic terminal regions are more responsive to palatable food in female vs. male rats [58]. These studies could serve as translational links to better understand sex differences in human food and drug-focused behaviors. For example, sweet-liking women, but not men, were more responsive to amphetamine [59] and women with binge eating disorder prefer sweets; an effect found to be related to the opiate system [60]. Finally, preference for high fat, sweet foods relates to obesity in women, not men [47].

## Conclusions

Female Long-Evans rats were more motivated to respond for sucrose and sucrose-paired cues than males. These results provide a robust model for further exploration of sex differences in sucrose reinforcement; the model may also facilitate research supporting a better understanding of addiction behaviors [7, 61].

## Supplementary Information

**Additional file 1:** Additional data from Experiments 4 and 5 including body weight corrected operant sucrose intake data.

## Data Availability

The data sets used and/or analyzed during the current study are available from the corresponding author upon reasonable request.
